# Five Actions to Strengthen the Nutrition and Dietetics Profession Into the Future: Perspectives From Australia and New Zealand

**DOI:** 10.1111/jhn.70064

**Published:** 2025-05-13

**Authors:** Rachel Boak, Claire Palermo, Eleanor J. Beck, Fiona Pelly, Clare Wall, Danielle Gallegos

**Affiliations:** ^1^ University of Melbourne Dental School, Faculty of Medicine, Dentistry and Health Sciences Victoria Melbourne Australia; ^2^ Faculty of Medicine, Nursing and Health Sciences Monash University Clayton Victoria Australia; ^3^ School of Health Sciences, Faculty of Medicine and Health University of New South Wales Sydney New South Wales Australia; ^4^ School of Medical, Indigenous and Health Sciences University of Wollongong Wollongong New South Wales Australia; ^5^ School of Health University of the Sunshine Coast Sippy Downs Queensland Australia; ^6^ Department of Nutrition and Dietetics, Faculty of Medical and Health Sciences University of Auckland Auckland New Zealand; ^7^ School of Exercise and Nutrition Sciences Queensland University of Technology Kelvin Grove Queensland Australia

**Keywords:** capabilities, diversity, forecasting, health professional organisations, professional identity, university education

## Abstract

**Introduction:**

Forecasting how workforces will meet projected challenges faced by future populations is essential to future‐proof professions. This paper aims to describe the specific actions required for nutrition professionals (nutrition scientists, nutritionists, dietitians) to realise the professions' workforce development vision of the future.

**Methods:**

A qualitative interpretive approach was employed. Individual interviews were conducted with nutrition leaders and external thought leaders. Focus groups were also conducted with experts within the nutrition and dietetics professions, academic dietetics educators and students/recent nutrition science and dietetics graduates (total sample *n* = 68). Key nutrition‐related issues and challenges, drivers for change and potential future roles of the profession were explored. This paper documents the actions required to achieve these outcomes. Data were analysed using a team‐based thematic analysis approach.

**Results:**

Analysis and interpretation of participant interviews identified five key actions to strengthen the profession: ‘Enhance and harness the diversity of the profession’; ‘Develop a cohesive profession with a strong identity’; ‘Decolonise teaching and practice’; ‘Retain science as the fundamental basis of the profession with trans‐systems knowledge integration’; and ‘Build opportunities for an agile nutrition workforce’.

**Conclusions:**

Future‐proofing the nutrition profession rests on mobilising current strengths and embracing growth and change. There will be confronting challenges for members of the profession and for a united profession. Individual professionals, professional associations, educators and education institutions need to work together to instigate change towards a more equitable future to support optimal health and wellbeing for individuals, communities and populations.

## Summary

- Five key actions to strengthen the profession were identified as: ‘Enhance and harness the diversity of the profession’; ‘Develop a cohesive profession with a strong identity’; ‘Decolonise teaching and practice’; ‘Retain science as the backbone of the profession but linked more effectively with the socioecological and environmental determinants of food and eating’; and ‘Build opportunities for an agile nutrition workforce’.
- Instigating change towards more equitable future health and wellbeing outcomes will bring confronting challenges for members of the profession, professional associations, educators and educational institutions.
- Future‐proofing the profession rests on mobilising current strengths, embracing growth and change, working together as one joined‐up and united nutrition and dietetics profession that is broadly conceptualised and is equipped to expand and diversify.

## 1 Introduction

Food and nutrition are critical for optimal health and wellbeing for individuals, communities and populations. Tertiary qualified nutrition professionals are ideally positioned to improve food and nutrition for all. For the purposes of this paper, nutrition professionals include those with formal university qualifications in biomedically derived evidence‐based nutrition, such as dietitians, nutrition scientists and public health nutritionists. These professionals play a critical role in building the evidence base, translating science into action and working with others to optimise multiple systems, including food, health and social systems, such as healthcare, the food system and social safety nets [1]. However, the profession is operating in increasingly complex and volatile contexts [2]. Climate change is disrupting the way food is grown and distributed [3]. Technology and communication are continuing to democratise knowledge with a plethora of ‘experts’ circulating nutrition information leading to a confused public. This is particularly relevant in the context of varied legal protections for the title of nutritionist and dietitian. These titles are not protected in all countries fuelling public distrust [4, 5]. Social movements, accelerated by the recent COVID pandemic, are disrupting work practices and highlighting institutionalised inequities that require dismantling [6, 7]. Previous work in the United Kingdom, United States and Australia/New Zealand has explored the future of their dietetics workforces, describing a growing demand for nutrition and dietetics professionals to work in community settings for chronic disease prevention and management, aged care health optimisation, personalised nutrition, nutrition sensitive food systems and technology/digital health [1, 8, 9, 10, 11, 12]. There is, however, a need to prioritise actions to ensure the workforce is equipped to expand and diversify.

In Australia and New Zealand, based on interview data with those inside and outside of the profession, Boak and colleagues identified six potential future‐focused roles for nutrition professionals. These included ‘food aficionados’; ‘diet optimisers’; ‘knowledge translators’; ‘equity champions’; ‘food systems activists’; and ‘change makers’ [1]. What these future‐focused roles will look like will vary across practice areas, workplace settings, political conditions and various other contextual factors. For example, if climate change is not mitigated, nutrition professionals may need to be championing equity in policy roles to coordinate and mobilise disaster food relief. Depending on the prevailing context, the role of food aficionado could see them as fusionists, blending creative works with food to create social connections. The research identifying these nutrition professional roles also identified 16 critical capabilities for optimising professional practice, to be developed through education and professional development programs [1]. Many of these, such as ‘curiosity’ and ‘critical thinking’, are already part of espoused graduate attributes and capabilities [13, 14, 15, 16]. Recently ‘lateral leadership’, the ability to work across sectors to engender transformation, has been identified as critical in the broad field of nutrition [17]. However, the competence of the breadth of the nutrition workforce to work across systems, to disrupt concepts of expertise, to draw on evidence beyond traditional biomedical science, and to demonstrate lateral leadership, remain largely unarticulated.

Without preparation for these new roles and harnessing of these capabilities, nutrition professionals will not necessarily be prepared to tackle changes and challenges across health, food and environments systems. Failure to prepare will limit the effectiveness of the profession, as other professional groups embrace these potential opportunities. The broader body of work on the future of the nutrition profession explored the roles and capabilities of nutrition professionals in the future; the aim of this paper is to describe the specific actions to be undertaken by nutrition professionals operationalise this workforce.

## 2 Materials and Methods

A qualitative interpretive approach was employed which embraced the diverse perspectives of all participants and their interactions with researchers. This manuscript builds on previously reported results of work to consider the future of ‘nutrition’ and nutrition professionals [1]. Where the previous work describes the roles and capabilities, this study describes the actions required to assist in this development as suggested by the participants. Researchers were experienced dietitians who shared diverse philosophical perspectives (scientific, pragmatic and interpretive) who regularly engaged in reflexive discussions throughout the study to embrace the multiple points of view of the research team. Ethics approval was provided by the relevant human research ethics committees of Queensland University of Technology (EC00171), The University of the Sunshine Coast (A201389), Monash University (24447), The University of Auckland, Latrobe University (2000000231) and University of Wollongong (2020/199).

The methods of the study have been previously described [1]. Purposive sampling was employed to identify a diverse and maximally varied sample of thought leaders, both internal and external to the profession. An initial sample consisted of 45 practitioners, researchers, interest group leaders, students, recent graduates, nutrition scientists, nutritionists and dietitians spread internationally, but with a focus in Australia and New Zealand. Experts within the nutrition profession (nutrition science, nutrition, dietetics), academic dietetics educators and students/recent graduates from both nutrition and dietetics were also invited to focus group discussions. Interview and focus group questions explored perspectives of current and future nutrition issues and how the workforce needs to be prepared to address them, and the future roles and skills required of the nutrition profession [1]. Data collection continued until the research team believed the data were rich and adequately captured a diversity of perspectives to answer the research questions.

We employed a team‐based approach to data analysis which facilitated the application of multiple views, meanings and interpretations. Thematic framework analysis [18] was used whereby line‐by‐line inductive coding of text was undertaken independently by three researchers (R.B., C.P., D.G.) on a subset of four different interviews each. These three researchers then met to compare and refine codes and descriptions and then apply the coding framework to each interview and focus group transcript. The dependability of the coding framework was checked by the other authors as they applied it to at least one of the interviews they undertook. All six interviewers completed contact summary sheets [19] for each interview and focus group. The contact summary sheet prompted interviewers to consider the main issues raised in the interview. It aimed to record salient, interesting, important, or illuminating points and take‐home messages. The sheets were completed after immersion in the interview transcript and were used to support data analysis. At the completion of coding, three researchers examined the data in the context of the research questions looking for patterns and frequencies across the data specifically on the research questions. The draft list of actions was presented to the research team for consideration and review, which facilitated discussions based on different research perspectives on the interpretation of the data. The actions were revised until all researchers were satisfied that the action reflected the data.

## 3 Results

A total of 33 individual interviews and nine focus groups were conducted, involving a total of 68 participants (Table 1). As previously reported (1) a further 10 people were invited to interview but either did not respond (*n* = 7) or declined (*n* = 3) due to lack of availability. Six of those invited who did not participate were within the nutrition profession and four were external. A majority (85%, *n* = 29) of participants lived in Australia and New Zealand, with the remaining participants from Canada (*n* = 2), United States (*n* = 1) and Europe (*n* = 1). Recruitment of individuals from the Pacific Islands proved unsuccessful. Most participants reported working in nutrition or dietetics (85%) and a number in academia (40%). Participants were from across a range of education, practice and work contexts, and were inside and outside of the nutrition profession.

**Table 1:** Characteristics of interview and focus group participants.

Area	Total participants
Thought leader—nutrition and dietetics professional^a^	25
Students^b^	12
Dietitians Australia members special interest groups^c^	10
Thought leader—external to nutrition and dietetics^d^	8
Dietetics educators/academics	6
Fellows dietitians Australia	4
Public health association of australia, food & nutrition interest group	3

^a^ Healthcare, Indigenous peoples' health and nutrition, Institutional foodservices, government bureaucrats, elite sports nutrition, academia, professional standards, curriculum and assessment in nutrition and dietetics, private practice, nutrition informatics, food industry.

^b^ Final year students currently enrolled in undergraduate or postgraduate nutrition and dietetics programs or nutrition science or human nutrition programs or recent graduates of these programs.

^c^ Food and environment, rehabilitation and aged care, food allergy and intolerance, eating disorders, public health and community nutrition, corporate, diabetes.

^d^ Systems scientists, International/global health, Indigenous peoples' health and nutrition, sociologies of education, health, food, government bureaucrats, food security, horticulture systems in developing countries, neuromusculoskeletal health and wellness.

Analysis and interpretation of participant interviews identified five key actions to strengthen the profession.

### 3.1 Action 1: ‘Enhance and Harness the Diversity of the Profession’

In the future, nutrition professionals will need to be critically aware of and actively engaged in shaping both their individual identity and that of the broad profession. The future profession must embrace and would be strengthened through diversity and inclusivity. A more diverse profession was described as a powerful resource, and as a source of learning, strength and knowledge.

Most participants described lack of diversity in the profession as an issue of concern or limitation for the profession. Participants recognised that the profession (particularly dietitians) in Australia and New Zealand is predominantly white, female and privileged. The profession is not diverse and does not typically reflect the communities and populations which the workforce serves. Areas directly and indirectly identified by participants that require a more diverse profile include race (actively working to increase the proportion of the profession who are of colour and who identify as Indigenous), gender (recruiting and retaining those who identify from a multiplicity of genders), class (working to recruit and retain the nutrition professionals from diverse socioeconomic and geographic backgrounds), and education (noting the profession will be strengthened by encouraging diverse pathways into the profession). Diversity extends across both those who have significant education but may have not have followed a traditional science pathway, to those who are from more diverse family education backgrounds, such as first in family to tertiary studies.

…about the diversity of the workforce, … we've been quite, you know, one type of culture. … the more we are diverse, the more we will tackle because it will be seen that those problems, those areas, those issues will be seen from the profession.

(FG8)

### 3.2 Action 2: ‘Develop a Cohesive Profession With a Strong Identity’

Participants described the need for a cohesive workforce delivering broad nutrition outcomes with a shared purpose of optimising health. This included embracing generalists and specialists across contexts and disciplines. For the nutrition profession this means constructing this as a joined‐up or consolidated workforce comprising a range of qualifications that are fit for purpose.

I think it's time for all those different parts of nutrition and dietetics to get together … this is a time for us to look at what opportunities that creates and recognise that we're much better doing it with strength and integrating it across the whole thing. Like all those little things that that least you know, they've all got a role for us. And if we did it together in an integrated and much more forceful way[it] will be much better than just all doing it in separate silos.

[INT011]

Such an approach could also facilitate greater community understanding of the roles of nutrition scientists, nutritionists and dietitians and is particularly relevant given the fluidity of the titles in many contexts.

I think that's one of our challenges, too, when you talk to people that work in the health department, they're always a little bit unclear on what a dietitian does, because we do span, you know, from prevention to treatment.

[FG8]

…nutrition science grads are in that weird space between people that have … inadequate nutrition qualifications, they got off like a two‐hour online course, and then you have clinically trained dietitians.

[INT019]

Participants explained that it was essential that this workforce is underpinned by robust credentialling, accreditation of education programs and supervision. The focus was on dietetic professionals but was salient for the entire nutrition profession. They suggested that education models needed to enable rather than disable movement across international borders and specialisation.

there was this push for globalisation, and also that discussion around globalisation of competencies and the moving workforce.

[FG1]

And I think that could be something universities could consider, is specialised areas that are provided by people that have had practice in that area and can put another level on it …

[INT024]

### 3.3 Action 3: ‘Decolonise Teaching and Practice’

The need for a diverse profession with a deep appreciation of the impact of power, poverty, trauma and racism on education, food choice and health was evident. This deep understanding was predicated on educators and professionals continuing to build their cultural safety and responsiveness. A proportion of participants voiced that curriculum content is dominated by predominantly white, industrialised, capitalist, heterosexual world views. In addition, educational institutions were reported to be influenced by social structures that promulgated an environment where rule compliance was promoted, and dissension discouraged.

… in a dietetics classroom, if you think about the privilege that resides in that space…. these are students that are in education, which costs money, presumably, but these are mostly white, upper middle‐class women who are being supported most of them, not all of them, to be learning, and they're being taught by white, privileged, educated, autonomous individuals, mostly women, again, in these institutions of just enormous privilege.

[INT007]

Participants reported that the academic structures in conjunction with curricula at every point determine who gets admitted, who thrives, who survives and who fails. This was reported to mirror the historical and economic realities of the unequal distribution of resources more broadly. These inequities follow students as they become professionals into the workplace. Participants spoke about disrupting the status quo in education, about integrating alternative ontologies which spoke to curriculum decolonisation. Specific to decolonisation of curricula, there are specific strategies that may be required. These include advocacy with Indigenous colleagues, education of nonindigenous educators and health professionals as allies, and explicit acknowledgement of systemic racism that may exist in institutions of both health and education.

How [will] the knowledge generators for our profession start to acknowledge that we need to rely more than just one way, one source of knowledge. So we need to start looking at different ways of knowing as legitimate and as being able to inform practices, so Indigenous ways of knowing, feminist ways of knowing

[INT 007]

### 3.4 Action 4: Retain Science as the Fundamental Basis of the Profession With Trans‐Systems Knowledge Integration

A strong science underpinning was described by participants as being critical for nutritionists and dietitians and a defining characteristic of the profession: ‘I still think that science is, is part of the backbone to our profession’ (FG7). Expanding the profession to integrate trans‐systems science elements was also articulated. This is relevant to professionals in nutrition, with or without additional dietetics qualifications. The science was also described as potentially constraining practice by contributing to a predominant biomedical approach focusing on biological systems (rather than a combination of social, economic and environmental) and on nutrients rather than foods. This has the potential to lead to the development of a mindset that was less able to take into consideration socio‐cultural and political systems thinking.

… we need to start looking at different ways of knowing as legitimate, and as being able to inform our practice. … positivist science has given us an incredible amount of very important knowledge and information on which this profession is based. But we need to move beyond and be more inclusive.

[INT007]

Participants suggested that nutrition professionals need to be able to continue to bridge the biological sciences with the social, economic, political systems and implementation sciences (a trans‐systems science approach) to be able to practically influence food choices, food behaviours and food systems. Participants argued that the future nutrition profession (inclusive of nutrition scientists, nutritionists and dietitians) needs the capacity to switch between biomedical, biological, social sciences and also to acknowledge and incorporate previously colonised and ostracised knowledge systems. depending on the outcomes and solutions required. The areas noted as needing to be incorporated, maintained or extended in education programs were communication and social media; dietary patterns; ethics; food and cooking; food systems (local and global); functional foods; Indigenous knowledges; intersectionality; nutrition‐sensitive agriculture; policy and political drivers; social practice; technology and artificial intelligence; cultural safety, particularly how to embrace it as a ‘lifelong practice’; and global and international health and social studies.

…there is a great awakening that food is actually, and eating, are social practices. You know, these are things that people do in a social space and the difficulty of pulling that off in a way that is satisfying and rewarding is increasingly difficult. …and so we're seeing this amazing coming together of a number of issues around food, the social context of food and what I'll call the cultural context of food.

[INT8]

It was not clear whether every individual requires all the skills described, but there was a recognition that a general understanding would allow all nutrition professionals to be aware of the breadth of approaches that may be required for translation of nutrition science to improve health outcomes.

### 3.5 Action 5: ‘Build Opportunities for an Agile Nutrition Workforce’

Participants suggested that the profession will need to be agile to adapt to new circumstances and to capitalise on opportunities that may emerge from a transdisciplinary focus. This would include building the skills of current nutrition professionals to increase specialisation in a particular area or enhance a current area of practice. It would also include enhancing professional skills to optimise movement across different systems of food, including, for example, broad health promotion and global food system and supply contexts, and more specific and localised food service provision contexts, such as in health or aged‐care institution settings. Developing the workforce to adapt to rapidly changing ways and contexts of providing care both within and external to traditional settings, such as hospital, primary care, institution, community or home, was reported as critical.

you train them as nutritionist, you give them the technical skill set. But do you supplement that with the additional skills to allow them to trade that in diverse operating environments? I see a lot of people who might be really technically good in the discipline, but they fall down because they can't modify the disciplinary activity to contextualize it to and to make it sympathetic to the operating environments that they work in.

[INT16]

It was noted that for those with dietetics qualifications, a refining of roles may be relevant with specialisation in some cases or relinquishing traditional work to different health or other professions (e.g., reduced focus on one‐to‐one treatment models in hospital settings, facilitating nutrition literacy of those charged with food provision in hospitals and greater extension to community or population) was described. An agile workforce would also be prepared and oriented to work across geographical boundaries, as a global workforce was also described.

Upskilling for all nutrition professionals, in areas that will enhance the ability to work across systems including but not limited to behaviour change and counselling, economics, user experience design, community development, technology, social media, marketing, business, agriculture and public policy. Each of these areas would attract a range of nutrition professionals who have trained, specialised or who have the proven experience to deliver the scope of work. Building the nutrition competence of other professions is also needed to create and extend an agile nutrition workforce. For example, educating medical, nursing and education professionals on the application of nutrition science or educating aged care staff on the use of nutrition screening tools. Participants predicted that the demand for a university‐qualified nutrition workforce would increase as would the need for other professions to be a part of the nutrition system.

Opportunities for discipline enrichment, such as learning a second language or actively encouraging the combining of nutrition degrees with other disciplines such as business, politics, economics and creative practice were also discussed. Such opportunities were reported to potentially increase the reach of the profession into non‐health sectors, such as agriculture and economics. This was perceived to lead to conceptualising these sectors as inclusive of roles for nutrition professionals, rather than as an encroachment on our ‘professional territory’.

… see the whole food system as from production or agriculture through to distribution, the supermarkets to eating and then waste, they only see it very much at the production end. So we have different worldviews, and we're going to need to overcome that. I think if we're to work together from a transdisciplinary perspective, they also see the concept of food security as quite different as well.

[INT15]

## 4 Discussion

To realise the vision of the future described by participants in this study, five specific actions to be undertaken by the profession were identified. Namely, to ‘enhance and harness the diversity of the profession’; ‘develop a cohesive profession with a strong identity’; ‘decolonise teaching and practice’; ‘Retain science as the fundamental basis of the profession with trans‐systems knowledge integration’; and ‘build opportunities for an agile nutrition workforce’. These findings provide further impetus and focus for how the profession can support and lead change.

Dietetics is a large workforce in the overall nutrition profession. In Australia, the dietetics profession is predominantly female and Australian‐born, with less than 0.5% identifying as Indigenous; however, other self‐identified markers of identity, such as ethnicity, dis/ability and LGBTQI+ status, remain unreported [20]. There is no available data for the nutrition profession with respect to identity markers. In New Zealand, research on dietitians in diabetes management and, more broadly, from the Ministry of Health have both defined the need to grow the workforce to support diversity [21, 22]. Similar inferences have been identified in Canada [23]. The finding in this study that a key action needed to strengthen and grow the profession is to ‘enhance and harness the diversity of the profession’ suggests that there is a pressing opportunity for professional and educational organisations to address the structural and socio‐cultural barriers that keep the profession demographically homogenised across factors such as race, body size and socioeconomic background. This aligns with ‘structural competency and awareness’ theories, and changes required in medical education and practice [24].

The nutrition profession is not alone in lacking a diverse workforce [25]. Lack of diversity in the health workforce, in particular, around race and ethnicity, is a known factor for limiting equitable access to healthcare in countries with a predominant white majority [26]. Cultures of conformity and homogeneity, competition, criticism and bullying, cohesion and conflict aversion, have been identified in Australian research on the dietetics profession [27]. It will only be through a concerted effort using a social justice lens to build critical cultural consciousness that a more diverse workforce can be created [28, 29]. Widespread use or understanding of models to address systemic, institutional, interpersonal and internal levels of privilege in all forms to enable critical allyship is needed [30]. In the nutrition profession, this would also include addressing diversity and equity issues not explicitly defined in the present study, such as ableism, neurodiversity and sexism.

One mechanism for tackling diversity is for education institutions to disrupt entry requirements to all nutrition programs but particularly to dietetics programs that currently predominantly privilege white middleclass entrants to the profession [20]. Notwithstanding institutional and political barriers, education institutions need to identify alternative pathways to the profession that maintain excellence and promote diversity. This includes identifying and removing barriers for Indigenous peoples to facilitate entry into the profession [31, 32, 33, 34]. Collaboration among professional associations, employers and education institutions is required to diversify the profession. Financial and other incentives for potential entrants to the profession, who may have had limited access due to the unconscious bias and structural barriers related to poverty, colour or gender, should be provided. It is important that these approaches are supported by curricula that articulate the principles and actions which will enhance the profession, namely ‘critical consciousness and emancipatory processes that transgress boundaries’, ‘anti‐oppression frameworks and a trauma‐informed lens’ and ‘decolonisation’ [28, 35, 36]. Definitions of these concepts are provided in Box 1. The curricula changes are part of an international call to ‘decolonise teaching and practice’ to enhance human rights and antiracism standpoints in nutrition, dietetics and health scholarship in national contexts, including Australia [32, 34] New Zealand [37], Canada [38] and the United Kingdom [39]. In colonised, settler countries such as Australia and New Zealand, which were invaded by a colonising force effectively dispossessing and decimating Indigenous populations, the nutrition profession can look to foundational scholarship in Indigenous education to advance reconciliation and also ground our teaching and learning in frameworks and policies such as the Aboriginal and Torres Strait Islander Health Curriculum Framework in Australia [40, 41, 42] and such as the Māori health models—Te Whare Tapa Whā in New Zealand [43].

Box 1 **Definition of key terms.**

*Anti‐oppression framework*

‘An anti‐oppression framework is one that seeks to undo the effects of oppression, oppose the roots of all forms of oppression, and to adopt an emancipatory approach to social change’ [25, p. 9].

*Trauma‐informed lens*

‘Trauma informed practice realizes the widespread impact of trauma, and pathways to recovery, recognizes symptoms of trauma in clients, participants, families, staff, systems, and in ourselves, responds to fully integrate trauma‐knowledge to improve and inform policies procedures and practices, and actively resists re‐traumatization’ [25, p. 9].

*Trauma*

‘The intergenerational and interpersonal trauma associated with colonialism and imperialism and the vast arrays of “isms” and phobias such as racism, sexism, ableism, classism, homophobia, and xenophobia, can be acknowledged and addressed in our everyday actions and in our policy proposals’ [25, p. 9].

*Decolonisation*

‘…employing approaches and methodologies that disrupt and reverse the ongoing exploitation and subjugation of people who have been marginalised, excluded and oppressed’ [25, p. 9].

*Critical consciousness*

‘Critical consciousness lies at the heart of working “inside out” to question perceptions of ourselves, of privilege and of the social and structural institutions that seek to maintain divisions in society. Critical consciousness highlights the need for a reflexive approach (that is not just thinking but also acting) to understand and change inequities in power and privilege’ [25, p. 8].

‘Critical consciousness involves a cycle of reflection and action consisting of three core processes: critical reflection, critical motivation and critical action. Critical reflection is the process of learning to examine social structures that serve to marginalise groups of people; critical motivation highlights the need to build capacity and commitment to track and address injustices; and critical action refers to intentionally engaging individually or collectively to dismantle injustices’ [42, p. 105].

*Intersectionality*

‘A theoretical approach that understands the interconnected nature of social categorisations—such as gender, sexual orientation, ethnicity, language, religion, class, socioeconomic status, gender identity, ability or age—which create overlapping and interdependent systems of discrimination or disadvantage for either an individual or group.’ [43, para 11].

A priority for professional organisations is strengthening their commitment to initiating and growing critical cultural consciousness [28, 44]. This may require challenging commonly held values and beliefs but is essential to ensure the ongoing disruption of the structures that seek to maintain inequities [44]. Education institutions teach emerging professionals the building blocks of critical cultural consciousness, but professional organisations are responsible for ensuring that it is a part of lifelong learning [44]. Australian organisations can learn from their New Zealand counterparts regarding processes and practices that actively work with Indigenous communities. Education can model courageous approaches to create culturally safe spaces for students to develop their critical cultural consciousness and to practice being innovators, disruptors, dissidents and discoverers without the fear of censure [28, 41]. Universities and educators have a key role to play in advocating for consistent, regular and timely reviews and updates to accreditation standards for education and practice in nutrition and dietetics to ensure that they are future focused, which is an evolving process that has progressed in recent times [13, 14].

The action to ‘keep science as the backbone of the profession’ suggests that the key onus of responsibility to expand curricula falls to education institutions. However, regulators will also have a role in ensuring that competencies and accreditation standards that inform curricular and pedagogical approaches to the education of the workforce reflect the integration of ways of knowing and other forms of knowledge. Professional organisations are encouraged to continue to develop a robust registration or credentialing system, as well as accreditation and recognition for dietitians, nutritionists, nutrition scientists and ancillary staff (e.g., nutrition assistants, chefs). This will require a flexibility in mindset that maintains quality and embraces uncertainty and diversity. As identified in education research [45], education institutions and regulators should continue to ensure that science underpins the education of the profession but that this is accompanied with highly developed critical and systems thinking skills. Existing scholarship has also cautioned that the focus on science may potentially constrain practice and intended practice outcomes by contributing to a focus on biological systems and on nutrients over whole foods [46, 47]. The results of this study emphasise that science will need to be supplemented with social, economic, political and implementation sciences to be able to practically influence food choices, food behaviours and food systems and for future professionals to actively dismantle structural barriers that serve to maintain social inequities. This will require more exposure to professionals in other systems, such as agriculture and economics, that may speak different ‘languages’. Finally, recent research has described the role of dietitians in educating and training future dietitians and other healthcare professionals and the need for embracing different approaches in this process [48, 49, 50].

This study highlights the opportunity for the profession to grow its current composition to enhance diversity, embed critical cultural consciousness in teaching and practice and build a stronger professional identity. This study also emphasises the priority for the profession to maintain its strong science foundations but also to build its ability to influence within and across systems, reconciling that other professions play a role in improving nutritional health and become integral to building their capacity to do so. Results of this study emphasise that the profession will grow and thrive if it can evolve beyond the simple characterisation of ‘dietitians’, ‘nutritionists’ or ‘nutrition scientists’ but at the same time have clarity around who is legally eligible to ensure public safety. Starting now, the Australian and New Zealand nutrition profession can actively embrace growth and change to be able to thrive in a future that may herald increasing ‘volatility, uncertainty, complexity and ambiguity’. A united joined‐up qualified nutrition profession will be essential, but like all change and growth, this process will be challenging. A joined‐up profession will be one that is streamlined and co‐ordinated, that transcends silos and nation‐state boundaries, addresses complexity and is committed to lateral leadership [51, 52, 53]. Further work and collaboration between professional associations, universities and sectors is required to realise concrete steps to operationalise the suggested actions. Future‐proofing will require individual professionals, professional associations, educators and education institutions to work together to instigate change. Courage has already been identified as a critical capability for the profession of nutrition and dietetics [1], and this courage is needed to lead our communities towards a better, healthier more equitable future.

## Author Contributions

All authors conceptualised the study. All authors collected interview data; R.B. collected focus group data. D.G., R.B. and C.P. analysed data with verification from all authors, and drafted the manuscript. All authors contributed to revising and editing manuscript.

## Ethics Statement

Ethics approval was provided by the relevant human research ethics committees of Queensland University of Technology (EC00171), The University of the Sunshine Coast (A201389), Monash University (24447), The University of Auckland, Latrobe University (2000000231) and University of Wollongong (2020/199).

## Conflicts of Interest

All authors at the time of the research were members of the Council of Deans Nutrition and Dietetics Australia New Zealand. The authors declare no other conflicts of interest.

### 1 Peer Review

The peer review history for this article is available at https://www.webofscience.com/api/gateway/wos/peer-review/10.1111/jhn.70064.

## Data Availability

The data that support the findings of this study are available on request from the corresponding author. The data are not publicly available due to privacy or ethical restrictions.
